# IHCH9033, a novel class I HDAC inhibitor, synergizes with FLT3 inhibitor and rescues quizartinib resistance in FLT3-ITD AML via enhancing DNA damage response

**DOI:** 10.1186/s40164-025-00605-y

**Published:** 2025-02-15

**Authors:** Mingyue Yao, Wenzhong Yan, Yafang Wang, Yu Zhao, Xiaowei Xu, Yujun Chen, Chengcheng Yu, Yingnian Li, Hualiang Jiang, Jie Shen, Jianjun Cheng, Chengying Xie

**Affiliations:** 1 Lingang Laboratory, 2380 Hechuan Road, Shanghai, 201101 China; 2 Shanghai Institute for Advanced Immunochemical Studies, ShanghaiTech University, Shanghai, 201210 China; 3 iHuman Institute, ShanghaiTech University, 393 Middle Huaxia Road, Shanghai, 201210 China; 4 School of Life Science and Technology, ShanghaiTech University, Shanghai, 201210 China; 5 Department of Hematology, Shanghai Jiao Tong University School of Medicine Affiliated Shanghai General Hospital, Shanghai, 200025 China; 6 Drug Discovery and Development Center, Shanghai Institute of Materia Medica, Chinese Academy of Sciences, Shanghai, 201203 China; 7 Department of Pharmacy, The SATCM Third Grade Laboratory of Traditional Chinese Medicine Preparations, Shuguang Hospital Affiliated to Shanghai University of Traditional Chinese Medicine, 528 Zhangheng Road, Shanghai, 201203 China

**Keywords:** Acute myeloid leukemia, HDAC inhibitor, FLT3-ITD mutation, DNA damage response, Synergistic effect, Drug resistance

## Abstract

**Background:**

Despite initial success with FLT3 inhibitors (FLT3is), outcomes for FLT3-ITD acute myeloid leukemia (AML) patients remain unsatisfactory, underscoring the need for more effective treatment options. Epigenetic modifications, such as histone acetylation, contribute to AML’s onset and persistence, advocating the potential for epigenetic therapies. However, the poor specificity of pan-histone deacetylase inhibitors (HDACis) leads to undesirable adverse effects, prompting the need for isoform-specific HDACis. This study aims to explore the antileukemic activities and mechanisms of IHCH9033, a novel class I HDACi, alone or combined with FLT3i in FLT3-ITD AML.

**Methods:**

The viability of AML cell lines and primary AML cells treated with HDACis alone or in combination with FLT3i was detected by MTT or CCK8 assay. Flow cytometry was utilized to examine cell apoptosis, cell cycle progression and ROS production. RNA sequencing analysis, RT-qPCR, western blotting, and co-immunoprecipitation assays were employed to elucidate the molecule mechanisms. The in vivo anti-leukemia efficacy was tested in xenografted mice models derived from FLT3-ITD cell lines and primary AML patients.

**Results:**

Here, we identified IHCH9033, a novel selective class I HDACi, which exhibited an increased antitumor effect in FLT3-ITD AML through effectively eliminating leukemia burden and overcoming resistance to FLT3i. Mechanically, IHCH9033 selectively inhibited DNA repair in FLT3-ITD AML cells, leading to the accumulation of DNA damage that eventually resulted in cell cycle arrest and apoptosis. Additionally, IHCH9033 induced HSP90 acetylation, FLT3 ubiquitination, and proteasomal degradation of FLT3, thereby inhibiting FLT3 downstream signaling. Notably, IHCH9033 maintained its potency in both FLT3i-resistant AML cell lines and primary-resistant patient samples, and exerted strong synergy with the FLT3i quizartinib, leading to tumor regression in FLT3-ITD/TKD AML xenografts. In patient-derived xenografts, the treatment with IHCH9033, both alone and in combination, led to nearly complete eradication of the AML burden, without significant adverse effects.

**Conclusions:**

Our study shows that IHCH9033, a novel class I HDACi with a desirable pharmacological profile, is a promising drug candidate for FLT3-ITD AML, and suggests a strategy of combining class I HDACis and FLT3is in AML clinical trials to increase efficacy and overcome resistance, thus potentially providing a curative treatment option.

**Supplementary Information:**

The online version contains supplementary material available at 10.1186/s40164-025-00605-y.

## Introduction

Acute myeloid leukemia (AML) is an aggressive malignancy characterized by the abnormal clonal expansion of immature myeloid precursors, which leads to impaired hematopoiesis and bone marrow failure [1, 2]. AML patients frequently display abnormal expression profiles, and the success of AML treatment largely relies on recurrent cytogenetic and genetic alterations. One prominent genetic molecular abnormality in AML, is the occurrence of internal tandem duplications (ITD) in the FMS-like tyrosine kinase 3 (FLT3) receptor gene, affecting 25–30% of patients. This mutation associated with an aggressive leukemic phenotype characterized by early relapse and poor treatment outcomes [3–5]. Despite recent therapeutic advances, the long-term survival rates for AML patients with FLT3-ITD remains very low [3, 6]. Several small molecule FLT3 inhibitors (FLT3is), including midostaurin and quizartinib, have been developed for treatment of FLT3-ITD AML, but their application as monotherapy has shown limited and transient clinical responses, with the majority of patients experiencing relapse and developing resistance shortly thereafter [7, 8]. The presence of CD34^+^ cells in AML is often associated with leukemic stem cells (LSCs) or precursor cells, which are able to initiate and sustain the development of leukemia [9–11]. Previous studies demonstrated that the persistence of this disease can also be attributed to residual LSC that are capable of evading cell death despite inhibited FLT3 kinase activity, leading to relapse upon discontinuation of tyrosine kinase inhibitors (TKI) therapy [12–14]. Therefore, it is pressing to develop new strategies that can effectively eliminate FLT3-ITD AML and overcome FLT3is resistance.

The dysregulation of epigenetic modifications is a critical hallmark of AML that significantly contributes to its pathogenesis. Modified epigenetic states and patterns in AML involve DNA methylation and altered histone modifications, particularly histone deacetylation, which impacts gene expression [15]. Histone deacetylases (HDACs) regulate histone deacetylation and their aberrant activity can lead to deregulated gene expression and protein function, thereby facilitating tumorigenesis and conferring drug resistance in leukemia [16]. Given these important roles of HDACs, pharmacological inhibition of HDACs has emerged as a primary focus of both preclinical and clinical investigations in leukemia over the past 20 years. Several HDAC inhibitors (HDACis), such as vorinostat, romidepsin, and belinostat, have received approval for the treatment of cutaneous T-cell lymphoma or peripheral T-cell lymphoma, with pracinostat also being granted orphan drug status for the treatment of AML [17, 18]. However, due to the high toxicity and limited efficacy of these pan-HDACis, their clinical utility is restricted. Consequently, the development of isoform-specific HDACis presents a rational strategy to enhance treatment efficacy leukemia by increasing potency and reducing toxicity.

Although there are currently no selective class I HDACis approved by Food and Drug Administration, several compounds, such as MGCD0103 are under going clinical trials, while MS-275 and tucidinostat have been approved in China for use as monotherapies for peripheral T-cell lymphoma (PTCL) or in combination with aromatase inhibitors for breast cancer patients. Although HDACi has shown moderate efficacy as single agent in early-phase clinical trials of AML, they exhibit more promised antitumor effects when utilized in combination therapies in both preclinical and clinical settings [19]. Tucidinostat has demonstrated potential in inhibiting cell proliferation, inducing G0/G1 cell cycle arrest and promoting cell apoptosis, as well as enhancing chemosensitivity, either standalone treatment or in combination with other agents in AML [20–22]. An option is to combine HDACi with TKI, exemplified by ongoing Phase I clinical trials investigating the combination of sorafenib and HDACi combination for refractory/relapsed AML patients (NCT01159301). Long et al. suggested that HDAC8 mediates TKI resistance and promotes leukemia maintenance in FLT3-ITD AML [23]. Furthermore, HDACi has been shown to induce proteasomal degradation of FLT3-ITD [24]. These findings encourage further exploration of the anti-leukemia effect achieved by combining class I selective HDACis with FLT3i, as well as their potential to overcome drug resistance.

IHCH9033 is a novel triazole-containing class I HDAC inhibitor developed by our research group. Previous studies have demonstrated its ability to inhibit tumor growth, enhance the anti-tumor immune responses, and synergy with immune checkpoint inhibitors [25]. In this study, we aimed to elucidate the antileukemic activities and mechanism of action of IHCH9033, both as a monotherapy and in combination with FLT3i in FLT3-ITD AML. Our findings indicate that IHCH9033 disrupted the DNA repair pathway, leading to the accumulation of DNA damage and consequent cell apoptosis, thereby demonstrating its antileukemic efficacy, even in TKI-resistant FLT3-ITD AML. These results suggest that combining class I HDAC and FLT3 inhibitors represents a promising strategy for treating FLT3-ITD AML and overcoming TKI resistance in both preclinical and clinical settings.

## Material and methods

### Compounds

**Fig. 1:**
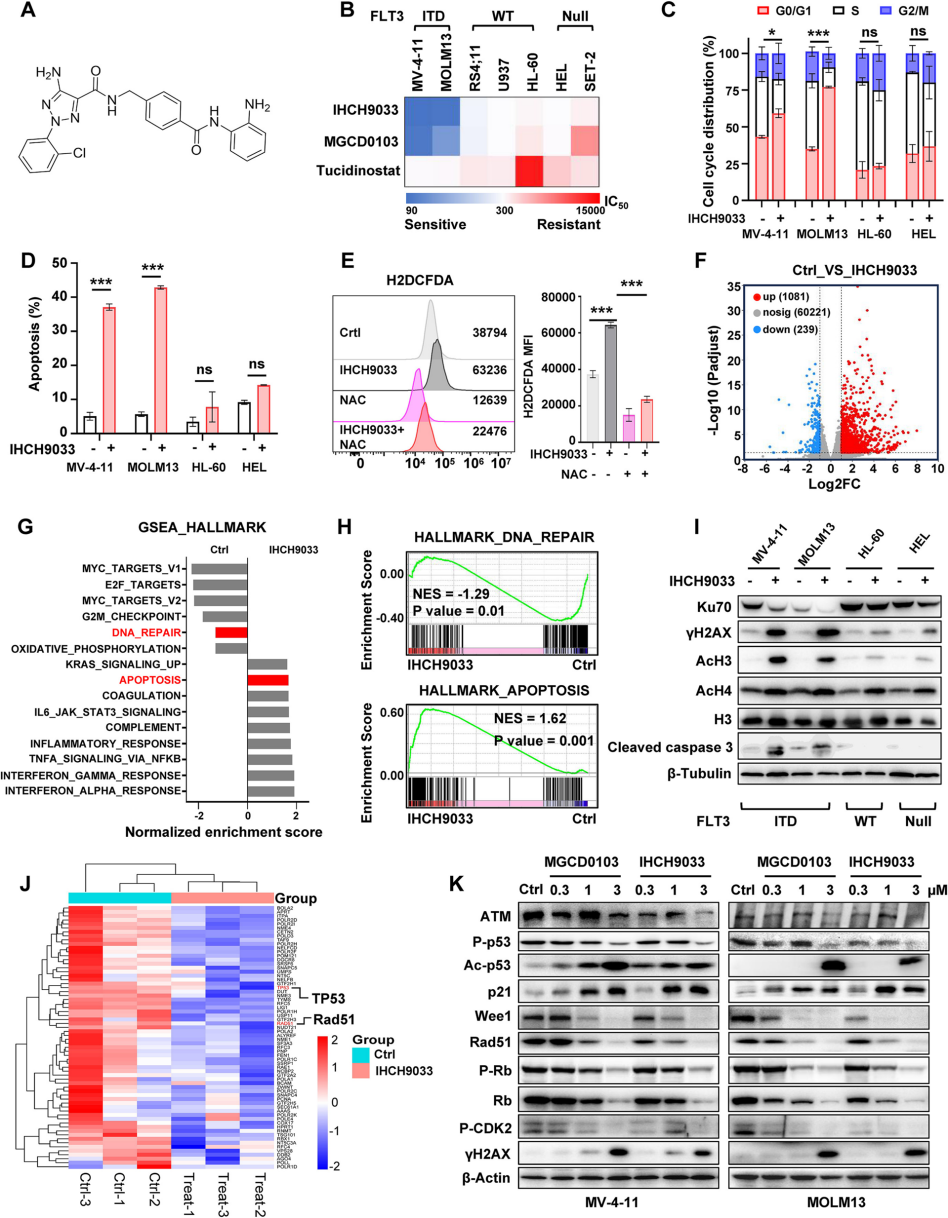
Class I HDACis exhibit preferential antitumor activity in FLT3-ITD AML cells by suppressing a broad DNA repair response. **a** Structure formula of IHCH9033. **b** The cytotoxicity of class I HDAC inhibitors (IHCH9033, MGCD0103 and Tucidinostat) in AML cell lines for 72 h were tested by MTT assay. **c** Cell cycle phase distributions were determined in MV-4-11, MOLM13, HL-60, and HEL cell lines after treated with 1 µM IHCH9033 for 24 h by staining the cells with propidium iodide and assessing their DNA content using flow cytometry. **d** Cell apoptosis induction was assessed in the AML cell lines by exposing the cells to the IHCH9033 (3 µM) for 24 h, followed by staining with annexin V-FITC/propidium iodide and subsequent analysis via flow cytometry. **e** The levels of reactive oxygen species (ROS) were detected using the fluorogenic dye H2DCFDA by flow cytometry in MOLM13 cells treated by IHCH9033, with or without pretreated NAC for 1.5 h (MFI: mean fluorescence intensity). **f** RNA-seq analysis was performed on MV-4-11 cells treated with IHCH9033 (0.5 µM) for 24 h, with volcano plots showing the different express genes (DEGs). **g** Bar plots illustrate the functionally enriched pathways associated with the significantly upregulated and downregulated genes (Ctrl vs. IHCH9033) in the HALLMARK cluster. **h** GSEA analysis on DNA_repair and apoptosis pathway in IHCH9033-treated MV-4-11 cells. **i** Western blot analyses assessed the protein levels of AcH3, AcH4, H3, cleaved caspase 3, Ku70 and γH2AX in the AML cell lines treated with IHCH9033 at 3 μM for 24 h. **j** Cluster analysis focused on DNA_repair pathway, indicating the downregulation of DNA repair genes. **k** The effect of class I HDACis on dose-course expressions of DNA repair proteins in FLT3-ITD cells. Data are mean ± SD. *P < 0.05; ***P < 0.001

IHCH9033 (Fig. 1a) was designed and synthesized in-house (Shanghai University of Science and Technology, Shanghai, China). Quizartinib, MGCD0103 and tucidinostat were purchased from MedChem Express (MCE; Monmouth Junction, NJ, USA). All compounds were dissolved in DMSO to prepare a 10 mM stock solution and were stored at − 80 °C for long-term storage as recommended.

### Cell lines and culture

The AML cell lines MV-4-11, RS4;11, U937 and HL-60 were purchased from the American Culture Collection Center (Manassas, VA, USA), while MOLM13, SET-2, and HEL were purchased from German Collection of Microorganisms and Cell Cultures (Braunschweig, Germany). All cells were grown in RPMI-1640 medium supplemented with 10% FBS, 1% penicillin/streptomycin, and maintained at 37 °C and 5% CO_2_ atmosphere. Cells were authenticated by short tandem repeat profiling (Geneing Biotechnologies Inc., Shanghai, China). Quizartinib-resistant cells (MV-4-11/quizartinib) were established by gradually increasing the concentration of quizartinib in the medium [26].

Peripheral blood or bone marrow samples were collected from patients with de novo AML following informed consent from each individual, with approval from the Ethics Committee of Shanghai Jiao Tong University School of Medicine Affiliated Shanghai General Hospital. The procedure was in accordance with the principles of the Declaration of Helsinki. Mononuclear blasts from healthy donors and AML patients were isolated using Lympholyte^®^-H (Cedarlane, CN), then exposed to ammonium-chloride-potassium lysis buffer (Thermo Fisher Scientific, MA, USA) to eliminate any residual red blood cells. The cells were cultured in RPMI-1640 medium supplemented with 10% FBS, and subsequently analyzed for cell viability and western blotting.

### Cell proliferation analysis

Cells were plated into 96-well flat-bottomed plates, and cultured in medium containing various concentrations of compounds at 37 °C in 5% CO_2_. Then, 3-(4,5-Dimethylthiazol-2-yl)-2,5-diphenyltetrazolium bromide (MTT; Sigma-Aldrich, MO, USA) or Cell Counting Kit-8 (CCK8; Meilune, Dalian, China) was added to per well and incubated at 37 °C. The effective dose of each drug that inhibited 50% growth (IC_50_) of these cell lines was calculated using GraphPad Prism 8. For the cell proliferation analysis of combination treatment, the HSA model synergy analysis was employed, and synergy scores were calculated using Synergyfinder 3.0 [27].

### Western blot analysis

Cells were lysed and whole-cell lysates were loaded onto SDS-PAGE gel. The proteins were then transferred to PVDF membranes (Millipore, MA, USA) and incubated with specific primary antibodies, followed by Horseradish peroxidase-conjugated secondary antibodies. Details of antibodies used were provided in Table S2, and the results were visualized using the Tanon Imaging System (Tanon, Shanghai, China).

### Co-immunoprecipitation (Co-IP)

After 12 h of incubation with IHCH9033, AML cells were collected and washed. The cells were then lysed in lysis buffer for 30 min at 4 °C. Protein supernatants were collected through centrifuge at 13,000 g for 10 min. The protein lysates were incubated with anti-FLT3 antibody and anti-acetylated lysine antibody overnight at 4 °C with constant rotation. The immunoprecipitates were incubated with protein A/G magnetic bead (MCE) for 4 h, then washed five times with lysis buffer and used for western blot analysis.

### ROS test assay

2′, 7′-dichloro-dihydro-fluorescein diacetate (H2DCFDA; Invitrogen, Carlsbad, CA, USA), a redox-sensitive fluorescent probe for superoxide, was used to detect intracellular ROS accumulation levels. After drug treatment, cells were incubated with 5 M H2DCFDA for 30 min, washed, and then analyzed for ROS generation by CytoFLEX flow cytometer (Beckman, Suzhou, China).

### Cell cycle assay

The distribution of AML cell cycle was assessed using propidium iodide (PI) immunofluorescence. Following the conclusion of drug treatment, AML cells were harvested and fixed in cold 70% ethanol overnight. Subsequently, RNase A digestion was performed, followed by PI staining at room temperature for 30 min. The samples were then analyzed using the CytoFLEX flow cytometer.

### Apoptosis assay

Cell apoptosis was determined using Annexin V-FITC/PI staining (Absin, Shanghai, China), following the manufacturer’s protocol, and detected via the CytoFLEX flow cytometer. To evaluate drug synergy, the combination index (CI) values were calculated using CalcuSyn Demo Version 2.0 software. A CI value greater than 1 signifies antagonism, a value less than 1 indicates synergy, while a value equal to 1 reflects an additive effect.

### RNA sequencing (RNA-seq) analysis

Total RNA was extracted from MV-4-11 parental and resistant cells using TRIzol^®^ Reagent (Invitrogen, Carlsbad, CA, USA), and sequenced by Majorbio (Shanghai, China), following the procedure demonstrated in a previous publication [28].

### RT-qPCR analysis

Following the manufacturer’s protocol, mRNA was extracted using TRIzol^®^ Reagent and reverse-transcribed for qPCR with PrimeScript^™^ RT reagent Kit (TAKARA, Osaka, Japan). SYBR Green PCR master mix (TAKARA) was applied for RT-qPCR on QuantStudio 6 (Thermo Fisher Scientific, MA, USA). The quantify of relative gene expression levels were analyzed by 2-ΔΔCT method. Primer sequences are provided in Table S3.

### In vivo study

Experimental procedures were conducted following the guidelines of the Animal Care and Use Committee of Shanghai Institute of Materia Medica, Chinese Academy of Sciences and Lingang Laboratory. Female nude mice aged 5–6 weeks (Vital River Laboratory Animal Technology, Beijing, China) were subcutaneously injected with 5 × 10^7^ AML cells. When tumors reached a volume of approximately 100 mm^3^, mice were randomly assigned and treated daily with vehicle, IHCH9033, MGCD0103, or tucidinostat. In the combined experiment, a daily oral administration of quizartinib in combination with IHCH9033 was conducted. The body weight and tumor sizes of the mice were assessed every 3 days, with tumor volumes determined using the formula: ½ × length × width^2^. Upon completion of the experiment, the mice were euthanized, and tumor samples were prepared for western blot analysis.

To generate patient-derived xenograft (PDX) models of AML, primary cells from bone marrow aspirates of AML patients were collected following approved protocols. Human CD34 positive (hCD34^+^) cells were isolated via magnetic bead isolation (MACS, Miltenyi Biotec), and then intravenously injected 1.2 × 10^6^ cells/mouse into female NOG mice (Vital River). Mice were sacrificed when they displayed signs of distress or disease, and bone marrow (BM) cells were collected for secondary transplantation (1.2 × 10^6^ cells/mouse). Disease progression was monitored by assessing the presence of human CD45 positive (hCD45^+^) or hCD34^+^ cells in the peripheral blood (PB) of the mice. Following confirmation of human leukemia engraftment in murine peripheral blood (0.5–2% human CD45^ +^ cells), treatment was initiated. Mice were administered by oral gavage with vehicle, quizartinib (10 mg/kg daily), IHCH9033 (60 mg/kg, every 2 days) or their combination.

### Statistical analyses

Data visualization and statistical analyses were conducted utilizing GraphPad Prism software. Statistical significance was assessed using the two-tailed unpaired Student’s *t*-test, with a *P* value less than 0.05 deemed significant across all experiments. Except where specified otherwise, all data presented as the mean ± standard deviation from a minimum of three independent experiments.

## Results

### IHCH9033 broadly suppresses DNA repair genes in FLT3-ITD AML cells, impairing DNA damage responses and leading to cell apoptosis

Firstly, we assessed the in vitro cytotoxicity of class I HDACis in AML cell lines with different FLT3 status. Here, IHCH9033, a novel triazole-containing HDAC inhibitor (Fig. 1a), and clinically relevant HDACis (MGCD0103 and tucidinostat) were chosen. The FLT3-ITD cells MV-4-11 and MOLM-13 showed significantly increased sensitivity to all these class I HDACis, when compared to FLT3- wild-type (WT) cells (RS4;11, U937 and HL60) and FLT3-Null cells (HEL and SET2) (Fig. 1b, Fig. S1A, Table S4). Accordingly, treatment with class I HDACis resulted in increased protein levels of AcH3 and AcH4 in FLT3-ITD cell lines (Fig. S1B). To delve deeper into the mechanisms by which class I HDACis impact cell viability, cell cycle analysis was performed. IHCH9033 treatment caused increased cell cycle arrest in the G0/G1 phase (Fig. 1c, Fig. S1C) and eventually induced apoptosis (Fig. 1d, Fig. S1D-E) in FLT3-ITD cells, whereas no notable alterations were observed in either FLT3-WT or FLT3-Null cells. It’s reported that FLT3-ITD cells accumulate ROS-induced DNA damage, causing increased repair errors that contribute to acquisition of additional mutations and aggressive nature of AML [29–31]. Here, we observed significant increase of endogenous ROS levels in FLT3-ITD AML cells after treated with IHCH9033, and pretreatment with the ROS scavenger N-Acetylcysteine (NAC) substantially reduced ROS production (Fig. 1e).

To investigate the selective mechanism underlying HDACi-induced cell death in FLT3-ITD cells, we performed RNA-seq analysis on MV-4-11 cells from both the control and IHCH9033-treated groups, revealing 1081 upregulated genes and 239 downregulated genes (Fig. 1f). Gene set enrichment analysis (GSEA) using HALLMARK datasets demonstrated that the DNA_REPAIR gene set was among the most significantly suppressed pathways, while APOPTOSIS gene set was one of the top increased pathways in IHCH9033-treated groups (Fig. 1g, h). Unrepaired or misrepaired DNA double-strand breaks (DSBs) have been shown to lead to apoptotic death or chromosomal damage [32, 33]. Accordingly, we found IHCH9033 significantly induced DNA damage in FLT3-ITD AML cells, as evidenced by increased protein levels of γH2AX (Fig. 1i), a well-established indicator of DSBs [34]. Moreover, we observed that IHCH9033 downregulated the protein expression of Ku70 in FLT3-ITD AML cells (Fig. 1i), a critical component of the error-prone nonhomologous end-joining (NHEJ) DNA repair pathway [35]. Results from RNA-seq analysis also revealed that IHCH9033 downregulated *TP53* and *RAD51* (Fig. 1j), crucial genes involved in another DNA repair pathway**—**homologous recombination (HR) [36]. Subsequently, western blot assays confirmed these findings, showing that treatment with IHCH9033 led to significant downregulation of the DNA damage sensor ATM [37, 38], along with its downstream DNA repair signals, including a marked decrease in p53 phosphorylation and increased γH2AX levels in FLT3-ITD AML cells (Fig. 1k). Additionally, IHCH9033 also activated p53 acetylation, enhanced its transcription activity, as indicated by the upregulation of p21 expression, and effectively inhibited the levels of Wee1 and Rad51 (Fig. 1k). Previous study has shown that following DNA damage, posttranslational modifications stabilize the p53 protein, enabling it to function as a transcription factor that upregulates genes promoting cell cycle exit and apoptosis in FLT3-ITD AML [39]. Accordingly, IHCH9033 inhibited phosphorylation levels of CDK2 and Rb, leading to G0/G1 cell cycle arrest. Summarily, the broad suppression of DNA repair genes by IHCH9033 in FLT3-ITD AML cells may impair cellular responses to DNA damage, ultimately resulting in the accumulation of DNA damage and subsequent cell apoptosis.

### IHCH9033 induces HSP90 acetylation and leads to the proteasomal degradation of FLT3 in FLT3-ITD AML cells

**Fig. 2:**
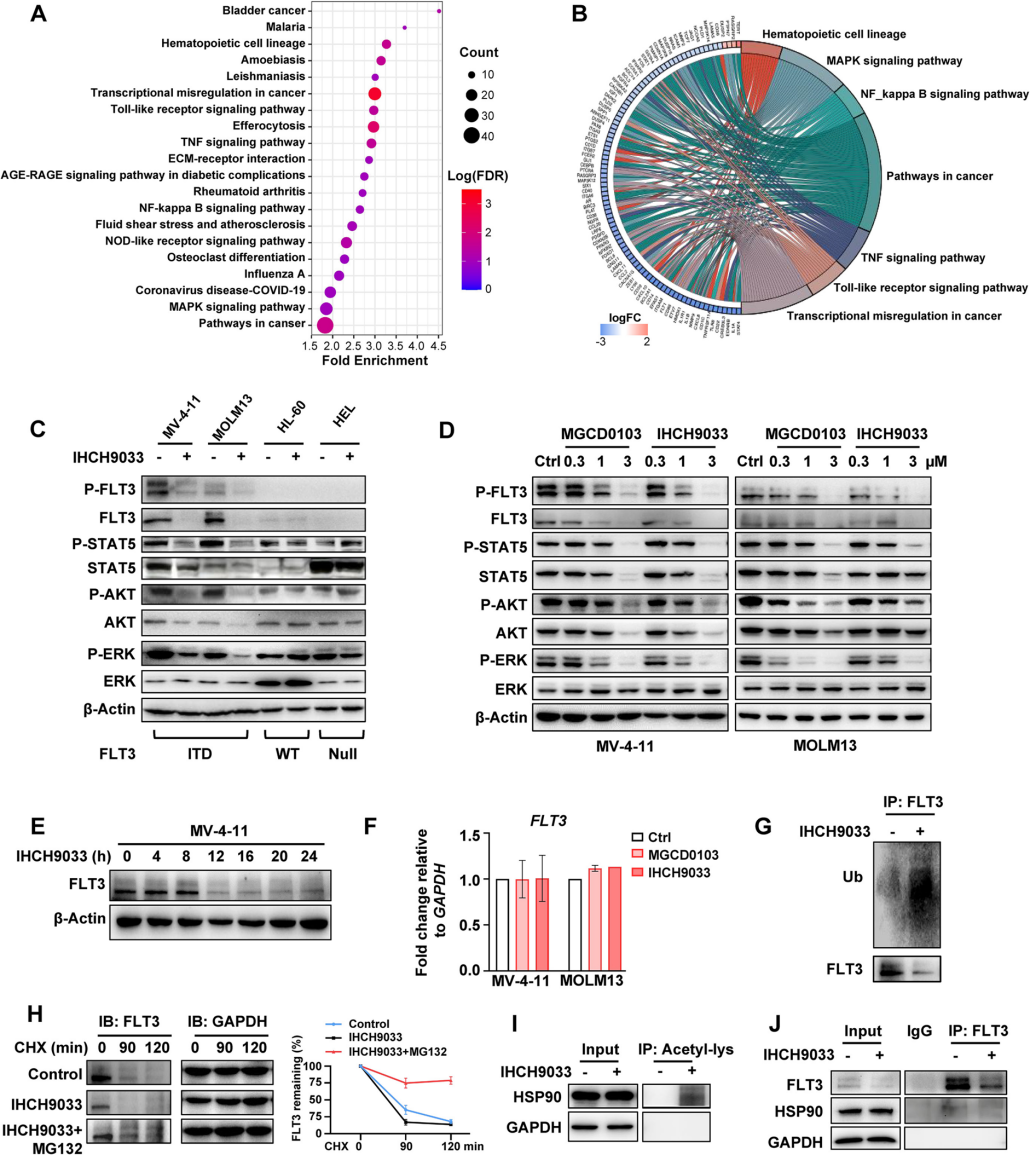
Class I HDACis induce FLT3 degradation through proteasome-dependent pathways in FLT3-ITD AML cells. **a**, **b** KEGG enrichment analysis of MV-4-11 cells treated with IHCH9033, displayed as a bubble plot (**a**) and a chord diagram (**b**). **c** The protein levels of FLT3 signaling pathway in MV-4-11, MOLM13, HL-60, and HEL cell lines were tested after incubation with 3 µM IHCH9033 for 24 h. **d** The effect of class I HDACis on dose-course expressions of FLT3 signaling pathway proteins in MV-4-11 and MOLM13 cell lines. **e** Time course treatment with IHCH9033 (3 µM) in MV-4-11 cells, with protein level of FLT3 measured by western blot. **f** The mRNA levels of FLT3 in MV-4-11 and MOLM13 cells were tested by RT-qPCR. **g** MV4-11 cells were treated with 3 µM IHCH9033 for 12 h. Protein lysates were immunoprecipitated with FLT3 antibody and then immunoblotted for ubiquitin (ub). **h** MV-4-11 cells were pretreated with IHCH9033 for 11 h, followed by the addition of MG132 for 1 h. Samples were collected after 20 μg/mL CHX treatment for 0, 90, and 120 min, respectively, with FLT3 protein level tested by western blot. The right panel was the FLT3 relative density quantified by Image J. **i**, **j** MV-4-11 cells were treated with 3 µM IHCH9033 for 12 h, after which cell extracts were prepared and subjected to immunoprecipitation analysis to detect the indicated proteins. Data are mean ± SD

HDACs are known to modulate the expression of non-histone proteins by influencing their acetylation balance [40]. KEGG analysis of the DEGs in MV-4-11 cells treated with IHCH9033 revealed prominent enrichment of pathways associated with RAS/RAF/MAPK signaling, which constitute the downstream signaling cascades of FLT3 (Fig. 2a, b). Subsequently, western blot analysis further showed that treatment with both IHCH9033 and MGCD0103 resulted in a significant decrease in the phosphorylation of FLT3 and its downstream targets, AKT, STAT5 and ERK, but this effect was observed only in FLT3-ITD AML cells (Fig. 2c, d). HDACis have been reported to cause degradation of FLT3-ITD [41], and accordingly, we found a marked reduction in the basal protein levels of FLT3 in a dose- and time-dependent manner following treatment with class I HDACi (Fig. 2d, e).

RT-qPCR analysis confirmed that the mRNA levels of FLT3 remained unchanged following treatment with either IHCH9033 or MGCD0103 in MV-4-11 and MOLM13 cells (Fig. 2f). However, IHCH9033 attenuated FLT3 expression level through the ubiquitination degradation pathway, and this effect could be rescued by proteasome inhibitor MG132 in FLT3-ITD AML cells (Fig. 2g, h). Previous research has shown that HDACis can bind to and induce acetylation of heat shock protein 90 (HSP90), leading to the inactivation of its chaperone activity and subsequent ubiquitination of the client proteins [24, 42, 43]. Here, we observed increased acetylation of HSP90 and fewer HSP90-FLT3 interactions in FLT3-ITD AML cells following treatment with IHCH9033 (Fig. 2i, j). Taken together, these findings suggest that inhibition of class I HDACs leads to HSP90 acetylation, FLT3 proteasomal degradation, and subsequent loss of FLT3 downstream signaling.

### IHCH9033 exerts potent antitumor efficacy in FLT3-ITD xenografted model and primary AML patient samples

**Fig. 3:**
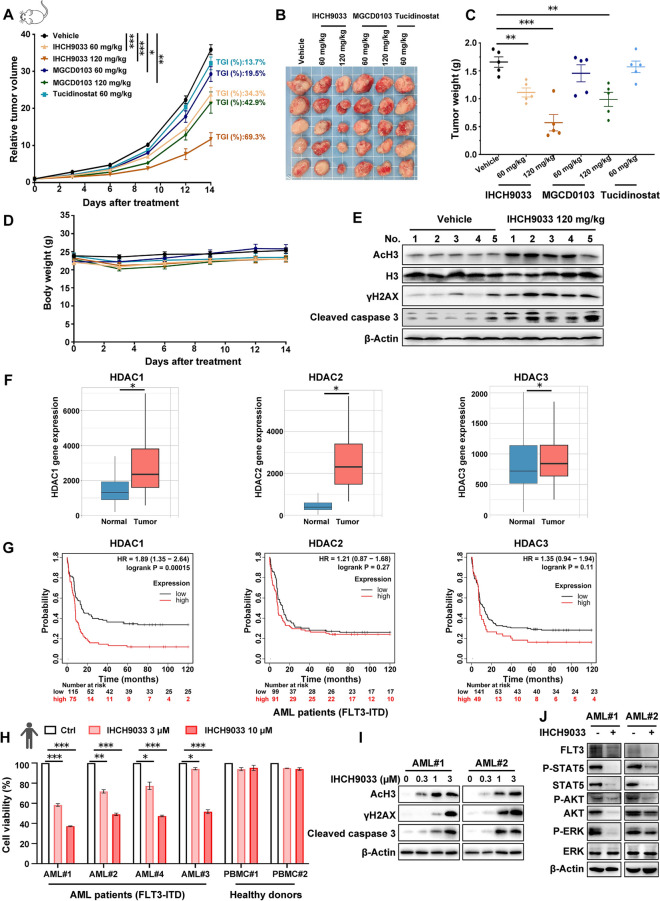
IHCH9033 exerts potent antitumor efficacy in FLT3-ITD xenografted model and primary AML patient samples. **a**–**d** Established MV-4-11 tumors were treated with vehicle, IHCH9033, MGCD0103, or tucidinostat at indicated doses in BALB/c nude mice. Tumor volume (**a**), tumor weight (**c**), and body weight (**d**) were monitored, and photograph of tumor tissue was shown in (**b**). **e** End-of-study tumors were harvested 3 h after the final dose, and protein levels of AcH3, AcH4, cleavage caspase 3 and γH2AX were analyzed by western blot. **f** Differential gene expression analysis of class I HDACs in AML and normal tissues from TNMplot database (http://www.tnmplot.com). **g** Survival analysis using Kaplan–Meier estimators and the log-rank test. Differences in survival times were analyzed by comparing FLT3-ITD AML patients with high vs. low levels of HDAC1/HDAC2/HDAC3. **h** Cell viability of healthy donor PBMC and primary AML blasts treated with the indicated dose of IHCH9033 for 24 h. **i**, **j** FLT3-ITD primary AML blasts were treated with IHCH9033 for 48 h, followed by western blot analysis. The vivo data are presented as mean ± SEM (n = 5), while other data are presented as mean ± SD, with n = 3. *P < 0.05; **P < 0.01; ***P < 0.001

Next, we investigated whether the in vitro effects of class I HDACis would translate to in vivo settings by testing this premise in mice engrafted with FLT3-ITD AML cells. In line with previous findings [20, 22], monotherapy with class I HDACis, specifically MGCD0103 or tucidinostat, showed limited efficacy in mice bearing MV-4-11 subcutaneous xenografts. However, IHCH9033 exhibited the most notable in vivo antitumor activity among them (Fig. 3a–c), aligning with its encouraging in vitro efficacy and favorable pharmacokinetic properties compared to MGCD0103 or tucidinostat [25]. Moreover, there were no significant changes in body weight across any of the treatment groups (Fig. 3d), except for the treatment of 120 mg/kg tucidinostat in our preliminary experiments, which resulted in a weight loss of over 20% in the mice (data not shown). Western blot analysis revealed that IHCH9033 treatment led to increased AcH3, along with elevated γH2AX and cleavage of caspase 3 in FLT3-ITD tumor tissues (Fig. 3e).

To investigate the potential impact of IHCH9033 treatment on AML patients, we executed comparable gene expression analysis of these class I HDAC members between AML tumor and normal tissues, revealing that HDAC1, HDAC2 and HDAC3 were notably overexpressed in AML (Fig. 3f). Kaplan–Meier survival analysis demonstrated that high levels of HDAC1 (HR 1.89, 95%CI 1.35–2.64) correlated with poorer overall survival (OS) in FLT3-ITD AML patients (Fig. 3g). Accordingly, our previous study has shown that IHCH9033, like MGCD0103 and tucidinostat, selectively inhibits HDAC1-3, with HDAC1 being the most sensitive target [25]. The antileukemic potential of IHCH9033 was further explored in primary AML cells derived from patients exhibiting variable FLT3-ITD mutant-to-wild allelic ratios (ARs) (Table S1), alongside peripheral blood mononuclear cells (PBMCs) from healthy donors. IHCH9033 had significant cytotoxicity in primary FLT3-ITD AML samples, while showing no such effects on normal PBMCs (Fig. 3h). Moreover, IHCH9033 significantly enhanced AcH3 level as well as DNA damage and cell apoptosis, as indicated by elevated γH2AX levels and caspase 3 cleavage (Fig. 3i), and it also reduced the phosphorylation of STAT5, ERK and AKT (Fig. 3j) in these FLT3-ITD AML patient samples. Together, these results suggest that IHCH9033 preferentially targets FLT3-ITD AML blasts while sparing normal hematopoietic cells. Our findings provide evidence supporting the preclinical assessment of HDACi for FLT3-ITD AML, and posit IHCH9033 as a promising candidate for potential clinical benefits.

### IHCH9033 synergizes with quizartinib to accumulate DNA damage and induce apoptosis in preclinical FLT3-ITD AML models

**Fig. 4:**
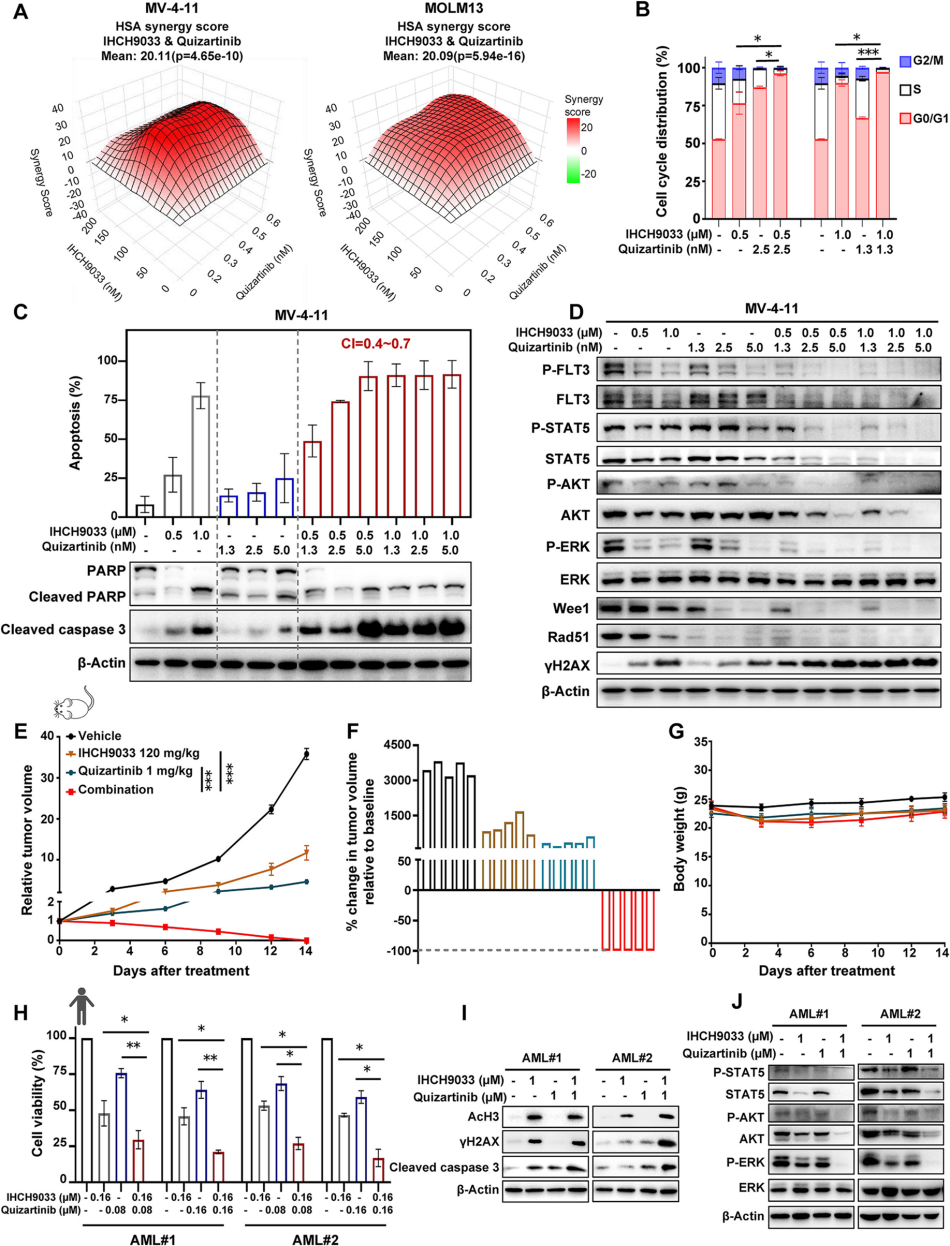
IHCH9033 synergizes with quizartinib in the treatment of FLT3-ITD AML. **a** The HSA model synergy analysis of IHCH9033 combined with quizartinib for 72 h in MV-4-11 and MOLM13 cells. Cells were treated with indicated concentration of IHCH9033 and quizartinib. Levels of cell proliferation were evaluated and results are presented as a HSA synergy score derived from the dose–response matrix. **b** MOLM13 cells were treated with IHCH9033 and quizartinib for 24 h, after which cell cycle distribution was analyzed by flow cytometry. **c** Cell apoptosis analysis of cells treated with IHCH9033 for 48 h, along with the calculation of CI by CalcuSyn (Upper panel), and the expression levels of protein related to cell apoptosis were detected by western blot (Lower panel). **d** The indicated protein levels after treatment of MV-4-11 and MOLM13 cells with IHCH9033 and quizartinib alone or in combination for 24 h were measured by western bloting. **e**–**g** MV-4-11 xenograft models were administered vehicle, IHCH9033 (120 mg/kg), quizartinib (1 mg/kg) or combination, with the tumor volumes measured accordingly (**e**), waterfall plot of tumor volume changes at the end of the study (**f**), along with mice’s body weight data (**g**). **h** FLT3-ITD primary AML blasts were treated with IHCH9033 and quizartinib alone or in combination for 5 days, after which cell viability was measured by CCK8 assay. **i**, **j** FLT3-ITD primary AML cells were treated with IHCH9033 and quizartinib alone or in combination for 48 h, followed by western blot analysis to detect the protein levels of specified antibodies. The vivo data are presented as mean ± SEM (n = 5), while other data are presented as mean ± SD, with n = 3. *P < 0.05; **P < 0.01; ***P <  0.001

Given the significant effect of class I HDAC inhibition on FLT3-ITD AML cells as mentioned above, we speculated that this could provide a rationale for combination therapy with FLT3i. Consequently, the combination of IHCH9033 with FLT3i quizartinib demonstrated significant synergistic cytotoxic effects in the FLT3-ITD AML cell lines MV-4-11 and MOLM-13 (Fig. 4a). Flow cytometry analysis further demonstrated that quizartinib markedly enhanced the ability of IHCH9033 to induce cell cycle arrest in the G0/G1 phase (Fig. 4b) and to promote caspase-dependent apoptosis in both FLT3-ITD AML cell lines (Fig. 4c, Fig. S2A). Furthermore, this combination decreased the expression of Wee1 and Rad51, along with an increased production of γH2AX, indicating that the combined regime further enhanced DNA damage and decreased the repair response in FLT3-ITD AML cells (Fig. 4d, Fig. S2B). Also, given the degrading effect of IHCH9033 on FLT3, the application of FLT3-ITD inhibitor potentiated the suppression of FLT3 phosphorylation and downstream signaling in the combination treatment regimens (Fig. 4d, Fig. S2B).

Subsequently, the in vivo antitumor efficacy of the combined HDAC and FLT3 inhibition was assessed in the MV-4-11 xenografted model. Notably, co-treatment demonstrated greater inhibition of tumor growth compared to individual monotherapies, with a tumor growth inhibition (TGI) value of 102.83% on day 14 after the start of treatment (Fig. 4e). Complete tumor regressions were observed in all mice within the cotreatment group (Fig. 4f), and this regimen was shown no significant weight loss observed in any of the treatment groups (Fig. 4g).

The previous study demonstrated that AML patients harboring high FLT3 mutant-to-wild type ratio (AR ≥ 0.5) exhibited poor outcomes [44]. We further analyzed the antileukemic effect of IHCH9033 combined with quizartinib in primary AML cells from two patients with high FLT3-ITD ARs (Table S1). Quizartinib alone exhibited limited inhibitory activity on the viability of primary human AML samples, unlike its effects observed in the FLT3-ITD cell lines MV-4-11 or MOLM13 (Fig. 4h, Fig. S2C). However, the combination of IHCH9033 and quizartinib significantly reduced cell viability (Fig. 4h) and exhibited increased levels of γH2AX and caspase-3 cleavage (Fig. 4i), indicating enhanced DNA damage and cell apoptosis in primary FLT3-ITD AML samples compared with either treatment alone. Corresponding to findings in AML cell lines, combination of IHCH9033 and quizartinib intensified the suppression of FLT3 signaling (Fig. 4j). Collectively, these findings indicate that combination of IHCH9033 and quizartinib exerted strong synergistic antitumor efficacy both in FLT3-ITD AML mouse xenografts and primary patient samples by inhibiting DNA repair processes and accumulating DNA damage.

### IHCH9033 overcomes resistance of FLT3-ITD AML to FLT3i treatment in vitro and in vivo

**Fig. 5:**
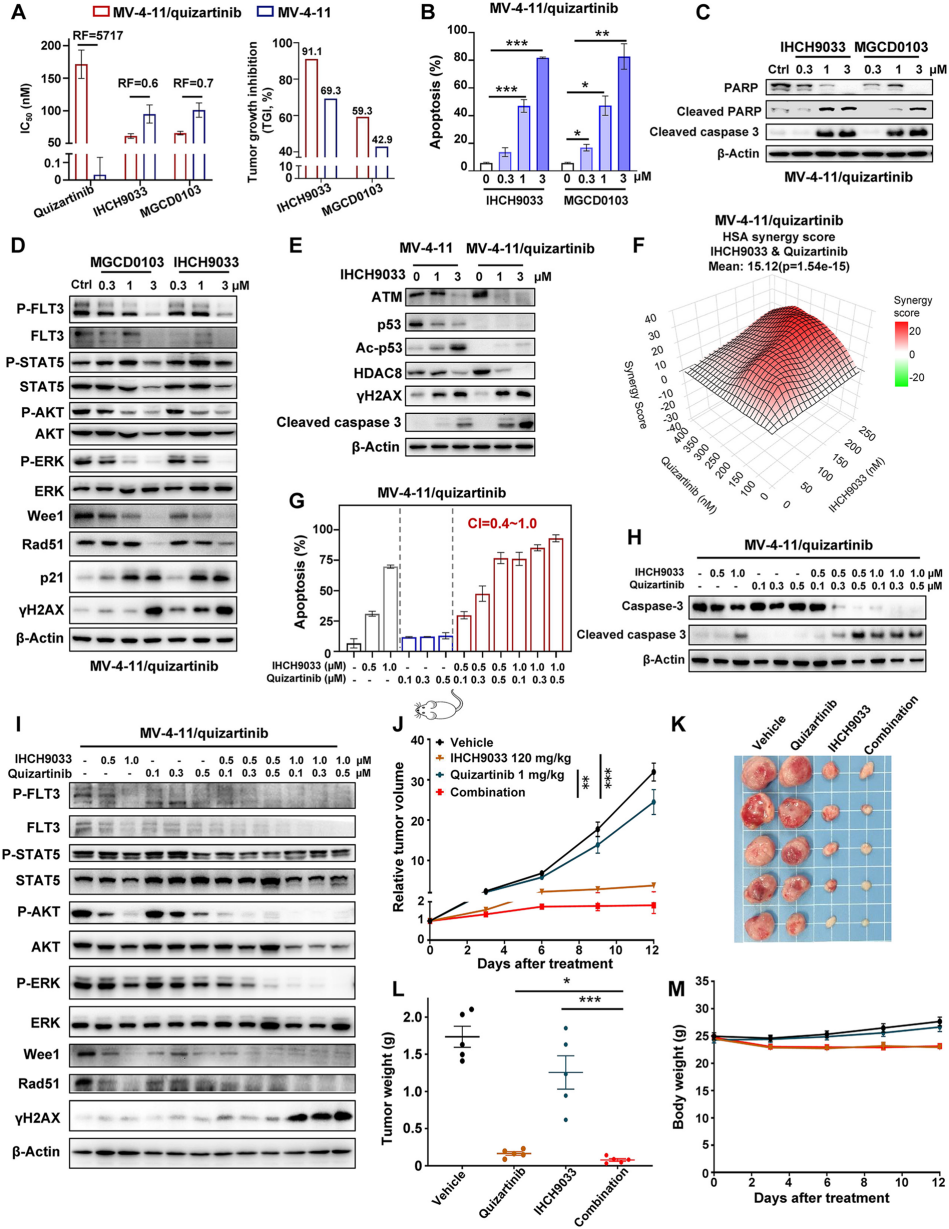
Class I HDACis mono-treatment or in combination with quizartinib overcomes the acquired resistance of FLT3-ITD AML to FLT3is. **a** The cytotoxicity of quizartinib, IHCH9033 and MGCD0103 in MV-4-11 parental and MV-4-11/quizartinib cells for 72 h were tested by MTT assay (Left panel). The efficacy of IHCH9033 (120 mg/kg), MGCD0103 (120 mg/kg) in MV-4-11 parental and MV-4-11/quizartinib xenografts (Right panel). RF, resisitance factor. **b** Cell apoptosis analysis of MV-4-11/quizartinib cells after treated with class I HDACis of indicated concentration for 48 h via flow cytometry (**b**) and western blot (**c**). **d** Western blot analysis of the FLT3 signaling and DNA damage and repair pathway in MV-4-11/quizartinib cells after treated with class I HDACis at the indicated concentration for 24 h. **e** Western blot analysis of MV-4-11 and MV-4-11/quizartinib cells after treated with IHCH9033 for 24 h. **f** The HSA model synergy analysis of IHCH9033 combined with quizartinib for 72 h in MV-4-11/quizartinib cells. Cells were treated with indicated concentration of IHCH9033 and quizartinib. Levels of cell proliferation were evaluated and results are presented as a HSA synergy score derived from the dose–response matrix. **g**, **h** Cell apoptosis analysis in MV-4-11/quizartinib cells after treated with IHCH9033 and quizartinib alone or in combination for 48 h via flow cytometry (**g**) and western blot (**h**). **i** The protein levels of the FLT3 signaling and DNA damage and repair pathway of MV-4-11/quizartinib cells after treated with IHCH9033 and quizartinib alone or in combination for 24 h were detected by western blot. **j**–**m** MV-4-11/quizartinib xenograft models were treated with vehicle, IHCH9033 (120 mg/kg), quizartinib (1 mg/kg) or their combination (n = 5). Tumor volume **j**, tumor weight (**l**) and body weight (**m**) were monitored, and photograph of tumor tissue was shown in (**k)**. The vivo data are presented as mean ± SEM (n = 5), while other data are presented as mean ± SD, with n = 3. *P < 0.05; **P < 0.01; ***P < 0.001

While several FLT3 TKIs have been developed for clinical use, their duration of response may not be sufficient, and resistance has also been reported concomitantly. To investigate the role of HDACis in leukemia maintenance and drug resistance, we generated the FLT3i-resistant AML cells MV-4-11/quizartinib harboring FLT3-ITD and F691L mutations [26], exhibiting a resistance factor (RF) of 5717 for quizartinib (Fig. 5a). Surprisingly, MV-4-11/quizartinib cells exhibited higher sensitivity to class I HDACi compared to the parental cells, evident from the lower IC_50_ values. Accordingly, MV-4-11/quizartinib xenografts demonstrated heightened sensitivity to IHCH9033, achieving a TGI of 91.1% at a dosage of 120 mg/kg, compared to 69.3% in the parental model. Comparable outcomes were noted for MGCD0103, although it’s in vivo antitumor activity was less potent than that of IHCH9033 (Fig. 5a). Furthermore, both IHCH9033 and MGCD0103 significantly induced apoptosis of MV-4-11/quizartinib cells, as indicated by the annexin V/PI assay results and western blot analysis (Fig. 5b, c).

Additionally, both IHCH9033 and MGCD0103 significantly decreased the phosphorylation levels of FLT3 signaling pathway in MV-4-11/quizartinib cells (Fig. 5d). Akin to the parent cells, IHCH9033 inhibited DNA repair proteins Wee1 and Rad51 expression, consequently inducing DNA damage as indicated by the increased levels of γH2AX in MV-4-11/quizartinib cells (Fig. 5d). As mentioned above, DNA repair defect was a crucial mechanism for targeting FLT3-ITD AML cells. Previous study has demonstrated that HDAC8 contributes to FLT3-ITD AML survival and TKI resistance by attenuating p53 activation [23]. Accordingly, western blot results demonstrated reduced protein levels of p53 and increased HDAC8 in MV-4-11/quizartinib cells compared to parental cells (Fig. 5e). Notably, HDAC8 were notably overexpressed in AML and Kaplan–Meier analysis demonstrated that high levels of HDAC8 (HR, 95%CI) were associated with poorer OS in FLT3-ITD AML patients (Fig. S3A-B). In addition, IHCH9033 significantly decreased ATM and increased caspase 3 cleavage in MV-4-11/quizartinib cells (Fig. 5e), further confirming the potential therapeutic vulnerabilities.

To further investigate the FLT3-dependent effect of HDACis, we generated 32D cells expressing FLT3-ITD or FLT3-ITD/TKD variants. As anticipated, FLT3-ITD 32D cells were sensitive to quizartinib treatment, while those expressing FLT3-ITD/F691L or FLT3-ITD/D835Y exhibited resistance. However, class I HDACis, including IHCH9033 and MGCD0103, indistinguishably suppressed cell proliferation and increased the AcH3 and AcH4 in all these FLT3-ITD or FLT3-ITD/TKD expressing 32D cells (Fig. S3C-D). Additionally, FLT3 degradation was observed following treatment with either IHCH9033 or MGCD0103 in all 32D cell lines transformed with FLT3-ITD/TKD (Fig. S3E).

Similar to parental cells, the cotreatment of IHCH9033 and FLT3i quizartinib exerted synergistic activity in antiproliferation and inducing apoptosis in MV-4-11/quizartinib resistant cells (Fig. 5f–h). As expected, the combination potentially increased γH2AX and reduced the levels of Wee1 and Rad51, as well as intensifying the suppression of FLT3 signaling pathway (Fig. 5i). Taken together, the combination of class I HDACi and FLT3i could synergistically inhibit FLT3 signaling and enhance DNA damage response, thus overcoming resistance to FLT3is.

We further validated the synergistic in vivo efficacy of the co-treating IHCH9033 and quizartinib in the MV-4-11/quizartinib tumor xenograft models (Fig. 5j–m). In alignment with the in vitro findings, MV4-11/quizartinib xenografts exhibited resistance to quizartinib, with a TGI of only 24.8% even at a dosage of 10 mg/kg. (Fig. 5j). However, the combination of IHCH9033 and quizartinib resulted in a remarkable additional reduction in tumor growth within the MV-4-11/quizartinib xenograft model (Fig. 5j–l), achieving a TGI of 97.3%, with no significant weight loss observed in any of the treatment groups (Fig. 5m). These results suggest that IHCH9033 can alter the fate of FLT3i-resistant AML cells on FLT3 inhibition, exhibiting significant potency against these cells and strong synergy with the FLT3i quizartinib characterized by increased levels of DNA damage and apoptosis and decreased levels of DNA repair proteins.

### IHCH9033 in combination with quizartinib effectively eradicates the leukemia burden in FLT3-ITD AML PDX models

**Fig. 6:**
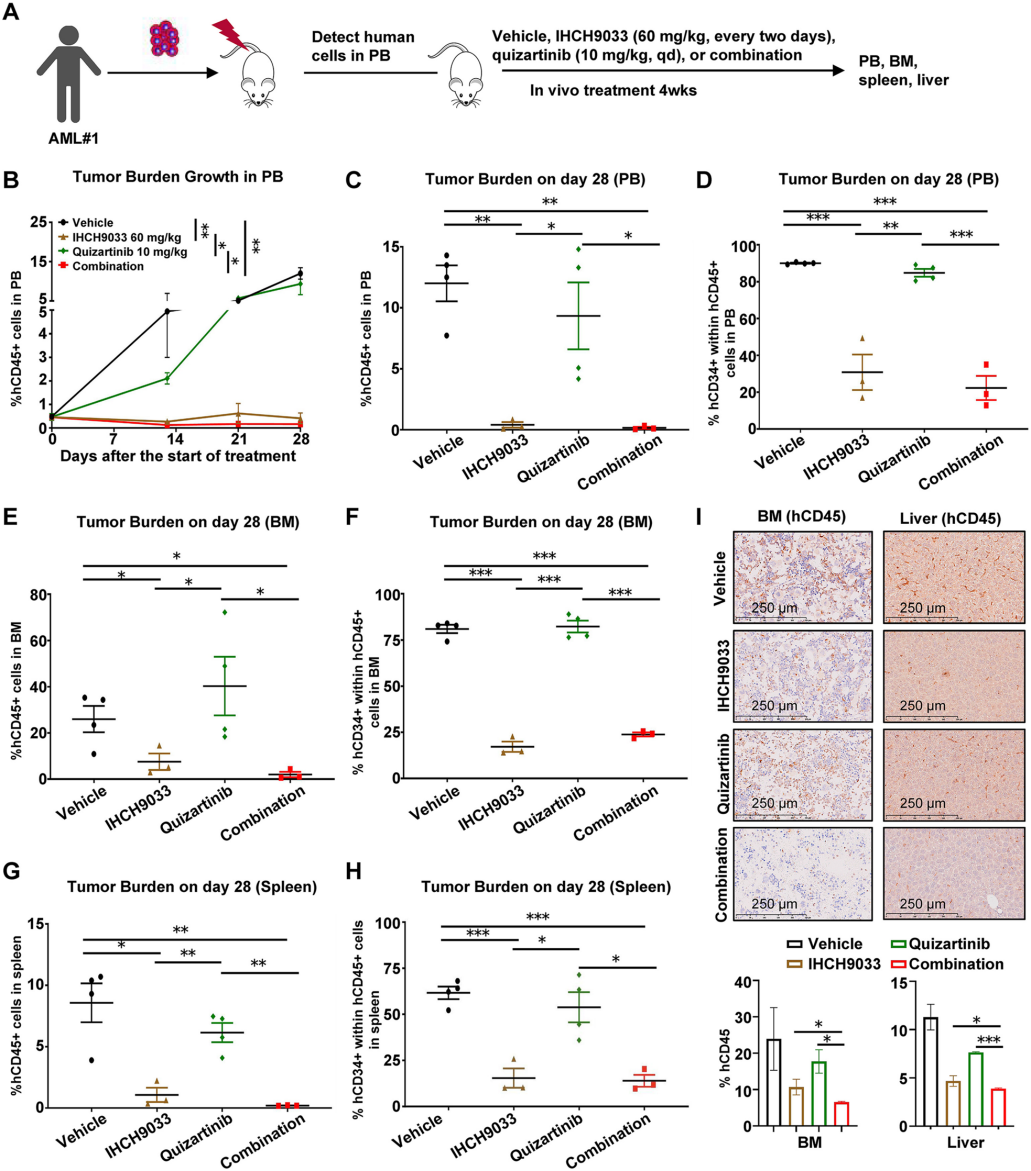
Antileukemic efficacy of IHCH9033 in combination with quizartinib in a FLT3-ITD AML PDX model. **a** NOG mice were engrafted with primary FLT3-ITD leukemia samples, after which groups of mice (n = 3–4) were treated with vehicle, IHCH9033 (60 mg/kg, every 2 days), quizartinib (10 mg/kg, qd), or their combination for 4 weeks. **b** The leukemic burden was monitored weekly by PB draws and quantitation of leukemic cells (human CD45-positive, hCD45 +) via flow cytometry on the indicated day. **c**, **d** PB engrafment on day 28, represented by the percentage of hCD45 + cells (**c**) and hCD34 + cells within the hCD45 + population (**d**). **e**, **f** BM engrafment on day 28, represented by the percentage of hCD45 + cells (**e**) and hCD34 + cells within the hCD45 + population (**f**). **g**, **h** Spleen engrafment on day 28, represented by the percentage of hCD45 + cells (**g**) and hCD34 + cells within the hCD45 + population (**h**). **i** BM samples or livers were collected on day 28 and IHC analysis using the indicated antibodies (n ≧ 3 mice/group); the scale bar represents 250 µm. Statistical analysis are shown below. Data are presented as mean ± SEM, *P < 0.05; **P < 0.01; ***P < 0.001

The clinical outcomes of older individuals, especially those with FLT3-ITD AML, remain unsatisfactory due to frequent comorbid conditions and, in particular, the genetic characteristics of the underlying disease. Combination strategies involving several targeted therapies may benefit additional older, less fit patients with AML [45]. To explore this, we evaluated the combined treatment of IHCH9033 and quizartinib in vivo using an AML PDX model. This model was established by intravenously injecting AML cells from an elderly patient with high ITD-AR (AML#1, Fig. 6a). The expression of hCD45^+^ served as a biomarker for leukemia burden, and its percentage was monitored. Given that HDACis are administered periodically to reduce toxicity and maintain therapeutic efficacy in clinical settings, an alternative dosing regimen for IHCH9033 at once every 2 days was employed. In contrast to the results from FLT3 -ITD MV-4-11 xenograft models, the administration of quizartinib at the dosage of 10 mg/kg showed minimal impact on the leukemia burden (Fig. 6b–i, Fig. S4). However, exclusive treatment with IHCH9033 (60 mg/kg, once every 2 days) significantly decreased the proportions of hCD45^+^ cells and/or hCD34^+^ CD45^+^ cells, signifying a reduced tumor burden within the bone marrow (BM), spleen, and peripheral blood (PB) of mice models (Fig. 6b–i). Furthermore, the combination led to nearly complete eradication of the AML burden. Strikingly, tumor infiltration of the BM and liver, as detected by IHC staining of hCD45, was significantly decreased in mice receiving the combination therapy (Fig. 6i). These results suggest that IHCH9033, either monotherapy or its combination with quizartinib, demonstrates notable efficacy for elderly AML patients with a high FLT3-ITD/WT ratio.

## Discussion

**Fig. 7:**
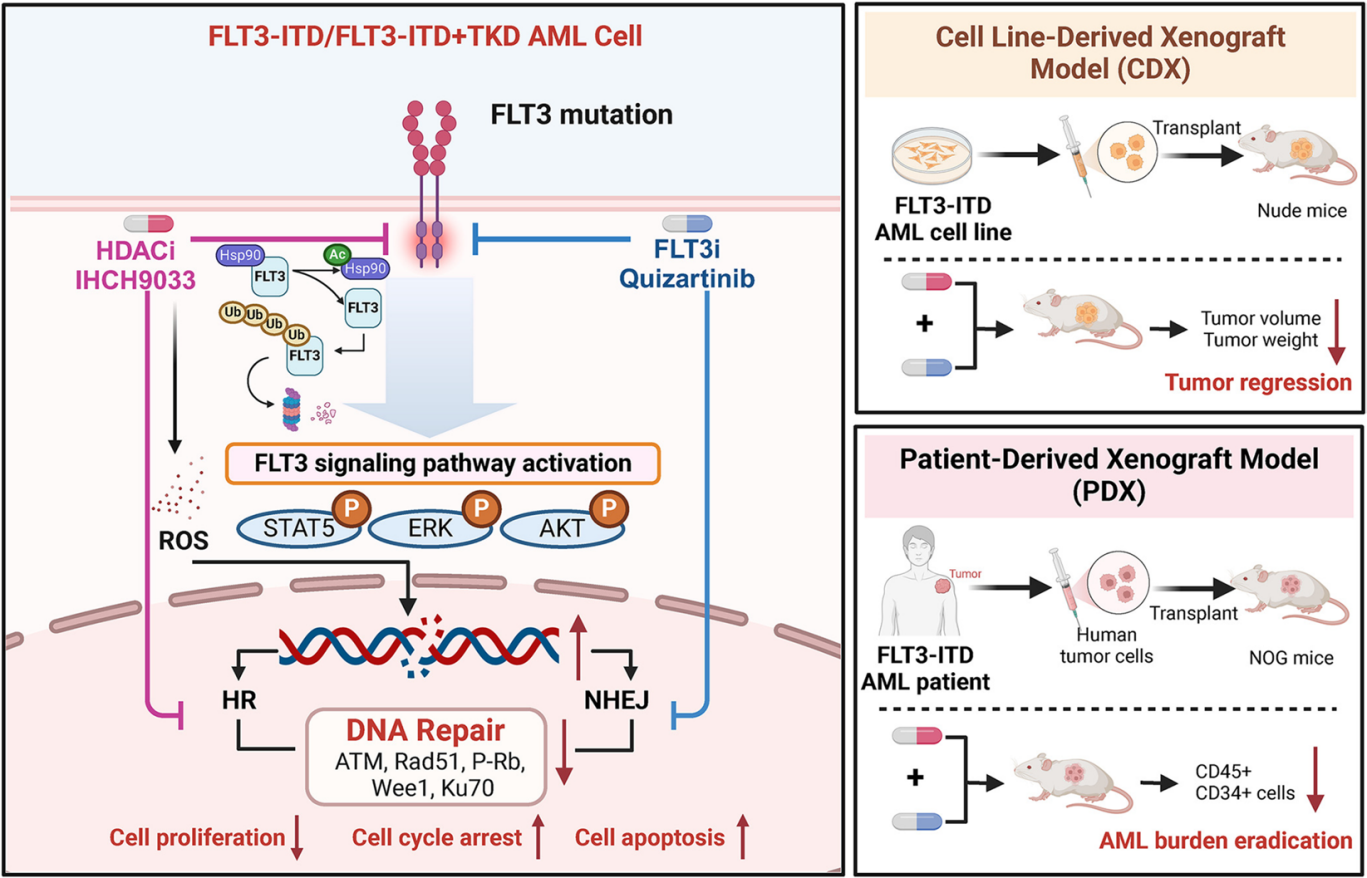
Proposed model showing the mechanism of combined HDAC and FLT3 inhibition. In FLT3-ITD/TKD AML cells, HDACi (IHCH9033) and FLT3i (quizartinib) synergistically inhibit FLT3 and DNA repair pathways, impairing the cellular DNA damage response, triggering cell apoptosis, and resulting in tumor regression in FLT3-ITD/TKD AML xenografts, as well as nearly complete eradication of AML burden in patients-derived xenografts. Created in https://BioRender.com

While epigenetic therapy for AML is still in its nascent stages, it shows immense potential and is advancing rapidly [46]. Several studies have revealed the limited impact of HDACis when used as a standalone treatment for AML [47]. However, combining HDACis with conventional chemotherapy or other epigenetic inhibitors has demonstrated some improvement in therapeutic efficacy, albeit with modest outcomes [48]. Preliminary clinical activity with MGCD0103, an isotype-specific HDACi, was observed in patients with refractory, relapsed acute leukemia and myelodysplastic syndrome [49]. This study provided compelling evidence of the potential therapeutic benefits of IHCH9033, a selective class I HDACi, either alone or in combination with FLT3i in preclinical models of FLT3-ITD AML. IHCH9033 demonstrated remarkable efficacy not only in TKI-resistant AML cells harboring FLT3 ITD/TKD mutations but also in primary-resistant patient samples. When combined with quizartinib, an FLT3i, it led to tumor regression in AML xenografts and nearly eradicated the AML burden in PDX models without significant side effects (Fig. 7).

The recurrence of cytogenetic and genetic alterations, particularly FLT3 mutations or duplications, significantly influences the efficacy of AML treatment [3]. FLT3-ITD mutations can elevate production of ROS, leading to increased DNA damage and inaccurate repair [30, 31, 50], which can contribute to a cycle of genomics instability and potentially create additional mutations that facilitate leukemic disease resistance [41, 51]. Recently, there has been a growing interest in the disruption of the DNA damage response with HDACis. For instance, HDACis downregulate genes related to checkpoints and DNA repair processes, including HR and NHEJ [50]. Here, we found that class I HDACis, including IHCH9033, selectively enhanced the ROS-induced DNA damage response, with evidence indicating that they broadly modulated DNA repair and damage signaling pathways in FLT3-ITD AML cells. This modulation may ultimately led to the accumulation of DNA damage beyond the threshold, which if not repaired in time, could potentially resulted in cell apoptosis.

HSP90, a cell chaperone protein [42], interacts with a wide range of client proteins, including pivotal oncogenic and antiapoptotic factors like FLT3, to prevent their ubiquitination and proteasomal degradation [52]. In addition, it has been documented that HDAC can regulate its ability to bind client proteins by regulating the acetylation activity of HSP90 [43]. This study demonstrated that IHCH9033 downregulated FLT3 protein levels in FLT3-ITD AML cells by promoting HSP90 acetylation. This alteration disrupts the HSP90-FLT3 interaction, leading to the degradation of FLT3 via the ubiquitination pathway. Also, HDACis degraded the FLT3 and suppressed downstream signaling, exhibiting similar activity in FLT3-ITD/F691L MV4-11/quizartinib or 32D cell lines transforming the FLT3-ITD/TKD mutations. The use of HDACi as monotherapy has shown moderate efficacy in early phase clinical trials for AML, while recent investigations indicate their potential to act synergistically with a variety of structurally and functionally diverse compounds, biologically active peptides, and novel immunotherapy [19, 53]. In the present study, the degradation effect on FLT3 itself, along with the significant anti-AML effects of IHCH9033 monotherapy, suggests that combining IHCH9033 with FLT3i could enhance the inhibition of FLT3 and downstream signaling, leading to the markedly synergistic antitumor activity. Notably, complete tumor regressions were observed in all mice treated with the combination of IHCH9033 with FLT3is, further underscoring the potential of this combination therapy in addressing FLT3-ITD AML.

Despite initial progress with FLT3is, even when combined with cytotoxic drugs, the outcomes of FLT3-ITD AML patients remain unsatisfactory, as these inhibitors primarily slow down the progression of the disease, rather than providing a complete cure [54]. They often fail to elicit a substantial initial response or are unable to sustain therapeutic benefits due to the emergence of secondary mutations, such as D835Y and F691L, in the FLT3-TKD during the therapy with FLT3i [54, 55]. These mutated cells exhibit greater resistance to FLT3i, such as quizartinib, compared to FLT3-ITD cells, resulting in relapse and poor patient prognosis, which is a representative mechanism of AML resistance observed clinically during quizartinib treatment [7, 54, 56]. Furthermore, the previous study has identified that the upregulation of class I HDAC member HDAC8 following FLT3 inhibition contributes to resistance and promotes leukemia maintenance [23]. Studies have reported that FLT3is can decrease the protein stability of p53, leading to the development of drug resistance [57]. The synergistic interaction between FLT3-ITD and p53 haploinsufficiency or loss contributes to enhanced proliferation, impaired differentiation, and inhibited apoptosis, ultimately facilitating the development of leukemia [58]. In this study, through transcriptome and protein level analysis, we demonstrated that MV-4-11/quizartinib resistant cells exhibited significant decreases in p53 expression and increased levels of HDAC8, suggesting potential molecular mechanisms underlying quizartinib resistance. Drug resistance in cancer cells is often accompanied by "fitness trade-offs", which subsequently expose new vulnerabilities in these resistant cells. One prevalent vulnerability identified in cells resistant to MAPK inhibitors is the increased production of ROS driven by hyperactive MAPK signaling [59]. Furthermore, our previous study revealed that FLT3i treatment did not decrease the phosphorylation of ERK in MV-4-11/quizartinib cell line [26]. Building on these earlier findings, both HDACi, such as IHCH9033 and MGCD0103, exhibited lower IC_50_ values and enhanced in vivo antitumor activity against this MV-4-11/quizartinib resistant model compared to the parental MV-4-11 AML, and demonstrated synergistic efficacy when combined with quizartinib. Thus, these findings indicate that HDACis could serve as a valuable treatment option, either alone or in combination with FLT3i, for AML patients, especially those with FLT3 secondary mutations. However, our studies did not exclude the possible contributions of other factors to the vulnerabilities in FLT3i-resistant AML cells, necessitating further investigation into the associated molecular mechanisms.

Targeting LSCs, a subset crucial for leukemogenesis and tumor recurrence, holds promise in improving clinical outcomes and potentially curing AML, but therapeutic options for eliminating LSCs are limited. Previous studies have shown that LSC-like cell lines and primary CD34^+^ AML progenitor cells exhibit limited sensitivity to monotherapy with HDACi [22]. The previous study reported that HDACs could be exploited to restore p53 activity and enhance targeting of LSCs in molecularly defined patient subsets [60]. Additionally, tucidinostat, by inhibiting HDAC3, in combination with chiglitazar, led to synergistic ferroptotic cell death in LSC-like cell lines and primary CD34^+^ LSCs, ultimately reducing tumor growth in a PDX model [61]. In this study, treatment with IHCH9033 alone or in combination with quizartinib significantly decreased cell viability and induced apoptosis in primary AML cells, while demonstrating minimal cytotoxicity on normal hematopoietic cells. Notably, IHCH9033 treatment significantly reduced the portion of hCD45^+^ and/or hCD45^+^hCD34^+^ in PB, BM and spleen in a primary FLT3-ITD AML PDX model, whereas quizartinib administration had minimal impact but significantly inhibited FLT3-ITD MV-4-11 xenograft tumors under the same treatment conditions. Furthermore, the combination of IHCH9033 and quizartinib effectively eliminated tumor burden in the AML PDX model. To mitigate toxicity and maintain effectiveness in clinical application, IHCH9033 was administered every 2 days as an alternative dosing regimen for HDACi. Correspondingly, this combination therapy was well tolerated, showing no impact on the body weight of the PDX mice and the absence of other therapeutic-related toxicities observed in vivo, further indicating its potential safety for future clinical trials. Given the selectivity inhibition of class I HDACs particularly HDAC3, and p53 activation by IHCH9033, along with its superior pharmacological properties, further investigation into its effects on AML LSCs is warranted, with future studies set to explore its activity both alone and in combination with quizartinib in this patient population.

Elderly AML patients, particularly those unfit for aggressive treatments like chemotherapy and stem cell transplantation, often have a poor prognosis with high rates of early mortality [62, 63]. Combination strategies, including the use of azacitidine alongside targeted therapies, hold promise for improving outcomes in this vulnerable population [45, 64]. In this study, we established an AML PDX model by intravenously injecting hCD34^+^ AML cells from an elderly patient with high FLT3-ITD-AR. The administration of IHCH9033 alone or in combination with quizartinib nearly eliminated leukemia burden in this PDX model, which was initially resistant to quizartinib. This findings indicated the potential efficacy of these treatments in future clinical trials, particularly for elderly AML patients who are unsuitable for more aggressive approaches. Although our comprehension of the epigenetic mechanisms involved in AML is still inadequate, this research provides an alternative option for AML patients harboring FLT3-ITD. Further investigation is needed to fully understand their specific influence on AML development and clinical assessments are needed to substantiate these conclusions. In addition, the outcomes for older individuals remain unsatisfactory, mainly due to the presence of comorbid conditions and, in particular, the genetic characteristics of the underlying disease [45].There is an urgent need to discover more potent inhibitors and to develop novel therapeutic approaches, including drug combination strategies, specifically for FLT3-ITD AML patients, in order to overcome current drug resistance.

## Conclusions

In summary, our study demonstrated that IHCH9033, a novel class I HDAC inhibitor, exhibited significant antitumor activity in FLT3-ITD AML, targeting both acquired-resistant AML cell lines and primary-resistant patient samples, through the modulation of DNA damage and repair response. Furthermore, the synergistic effect of IHCH9033 and quizartinib was mechanistically linked to enhanced targeted modulation of FLT3 and downstream signaling pathways. These findings provide a foundational rationale for the combination of class I HDACis with FLT3is in the treatment of AML, and suggest a promising new therapeutic strategy for addressing FLT3i-resistant AML in clinical settings.

## Supplementary Information

Additional file 1.

## Data Availability

No datasets were generated or analysed during the current study.

## Abbreviations

AML: Acute myeloid leukemia
CI: Combination index
FLT3: FMS-like tyrosine kinase 3
FLT3is: FLT3 inhibitors
GSEA: Gene set enrichment analysis
HDACs: Histone deacetylases
HDACis: HDAC inhibitors
HSP90: Heat shock protein 90
ITD: Internal tandem duplications
LSCs: Leukemic stem cells
NAC: N-Acetylcysteine
NHEJ: Nonhomologous end-joining
OS: Overall survival
PBMCs: Peripheral blood mononuclear cells
PDX: Patient-derived xenografts
PI: Propidium iodide
TKD: Tyrosine kinase domain
TKI: Tyrosine kinase inhibitors
TGI: Tumor Growth Inhibition
