# Supplementary material for: IHCH9033, a novel class I HDAC inhibitor, synergizes with FLT3 inhibitor and rescues quizartinib resistance in FLT3-ITD AML via enhancing DNA damage response
Source: Exp Hematol Oncol. 2025 Feb 15;14:15. doi: 10.1186/s40164-025-00605-y (PMC11829435; doi:10.1186/s40164-025-00605-y)
Supplement: Supplementary file 1 — Additional file 1. [file 40164_2025_605_MOESM1_ESM.docx]

**SUPPLEMENTARY INFORMATION**

**IHCH9033, a novel** **class Ⅰ HDAC inhibitor, synergizes with FLT3 inhibitor and rescues quizartinib resistance in FLT3-ITD AML via enhancing DNA damage response**

Mingyue Yao^1,2#^, Wenzhong Yan^3#^, Yafang Wang^2^, Yu Zhao^2,4^, Xiaowei Xu^5^, Yujun Chen^2,4^, Chengcheng Yu^1^, Yinnian Li^1^, Hualiang Jiang^2,4,6^, Jie Shen^7^*, Jianjun Cheng^3,4*^, Chengying Xie^1, 2,4*^

**Affiliations:**

^1^Lingang Laboratory, 319 Yueyang Road, Shanghai 200031, China

^2^Shanghai Institute for Advanced Immunochemical Studies, ShanghaiTech University, Shanghai 201210, China

^3^iHuman Institute, ShanghaiTech University, Shanghai 201210, China

^4^School of Life Science and Technology, ShanghaiTech University, Shanghai 201210, China

^5^Department of Hematology, Shanghai Jiao Tong University School of Medicine Affiliated Shanghai General Hospital, Shanghai 200025, China

^6^Drug Discovery and Development Center, Shanghai Institute of Materia Medica, Chinese Academy of Sciences, Shanghai 201203, China

^7^Department of Pharmacy, The SATCM Third Grade Laboratory of Traditional Chinese Medicine Preparations, Shuguang Hospital Affiliated to Shanghai University of Traditional Chinese Medicine, Shanghai 201203, China

^#^ These authors contributed equally.

*Corresponding authors.

Supporting tables and figures

Table S1-S4

Fig. S1-S4

**Table S1. Clinical and biological features of primary AML patient samples.**

| **AML Patient** | **Age** | **Gender** | **ITD/WT** |
| --- | --- | --- | --- |
| 1 | 63 | Male | 0.81 |
| 2 | 32 | Male | 1.03 |
| 3 | 57 | Male | 0.002 |
| 4 | 66 | Male | 0.575 |

**Table S2. Antibodies table**

| **Antibody** | **Source** | **Identifier** |
| --- | --- | --- |
| Ku70 | ABclonal | Cat# A11223 |
| γH2AX | Cell Signaling Technologies | Cat# 2577 |
| Acetylated histone H3 (AcH3) | Cell Signaling Technologies | Cat# 4499 |
| Acetylated histone H4 (AcH4) | Cell Signaling Technologies | Cat# 13534 |
| Histone H3 (H3) | Cell Signaling Technologies | Cat# 4499 |
| Histone H4 (H4) | Cell Signaling Technologies | Cat# 13919 |
| cleaved caspase 3 | Cell Signaling Technologies | Cat# 9664 |
| β-Tubulin | Cell Signaling Technologies | Cat# 2128 |
| ATM | Cell Signaling Technologies | Cat# 2873 |
| P-p53 | Cell Signaling Technologies | Cat# 2676 |
| Acetylated p53(Ac-p53) | Cell Signaling Technologies | Cat# 2525 |
| p21 | Cell Signaling Technologies | Cat# 2947 |
| Wee1 | Cell Signaling Technologies | Cat# 13084 |
| Rad51 | Cell Signaling Technologies | Cat# 8875 |
| P-Rb | Selleck | Cat# F2220 |
| Rb | Cell Signaling Technologies | Cat# 9313 |
| P-CDK2 | Cell Signaling Technologies | Cat# 2561 |
| P-FLT3 | Cell Signaling Technologies | Cat# 3464 |
| β-Actin | Cell Signaling Technologies | Cat# 3700 |
| FLT3 | Cell Signaling Technologies | Cat# 3462 |
| P-STAT5 | Cell Signaling Technologies | Cat# 4322 |
| STAT5 | Cell Signaling Technologies | Cat# 25656 |
| P-AKT | Cell Signaling Technologies | Cat# 4060 |
| AKT | Cell Signaling Technologies | Cat# 13038 |
| P-ERK | Cell Signaling Technologies | Cat# 4370 |
| ERK | Cell Signaling Technologies | Cat# 4695 |
| HSP90 | Cell Signaling Technologies | Cat# 4877 |
| GAPDH | Cell Signaling Technologies | Cat# 5174 |
| PARP | Cell Signaling Technologies | Cat# 9532 |
| HDAC8 | Cell Signaling Technologies | Cat# 66042 |
| Acetylated lysine | Cell Signaling Technologies | Cat# 9441 |

**Table S3. Sequences in RT-qPCR.**

| **Gene** | **Forward (5’-3’)** | **Reverse (5’-3’)** |
| --- | --- | --- |
| *FLT3* | CGAGGAGGGCAACTACTTTGAGATG | TGACTGGGATGCTTTGAAGAGGAAC |
| *GAPDH* | GACATCAAGAAGGGGTGAA | TGTCATACCAGGAAATGAGC |

**Table S4. Antiproliferative activities of class I HDAC inhibitors in AML cell lines.**

| **Cell lines** | **FLT3** | **IC_50_ (nM, Mean ± SD)** | | |
| --- | --- | --- | --- | --- |
|  |  | **IHCH9033** | **MGCD0103** | **Tucidinostat** |
| MV-4-11 | FLT3-ITD | 95 ± 14 | 101 ± 11 | 515 ± 62 |
| MOLM13 | FLT3-ITD | 99 ± 19 | 152 ± 22 | 598 ± 11 |
| RS4;11 | FLT3-WT | 301 ± 27 | 302 ± 1 | 1319 ± 121 |
| U937 | FLT3-WT | 289 ± 124 | 286 ± 50 | 1524 ± 308 |
| HL-60 | FLT3-WT | 722 ± 14 | 1573 ± 10 | 13496 ± 873 |
| HEL | FLT3-Null | 378 ± 42 | 419 ± 9 | 3293 ± 769 |
| SET-2 | FLT3-Null | 1027 ± 53 | 6588 ± 321 | 1891 ± 78 |

**
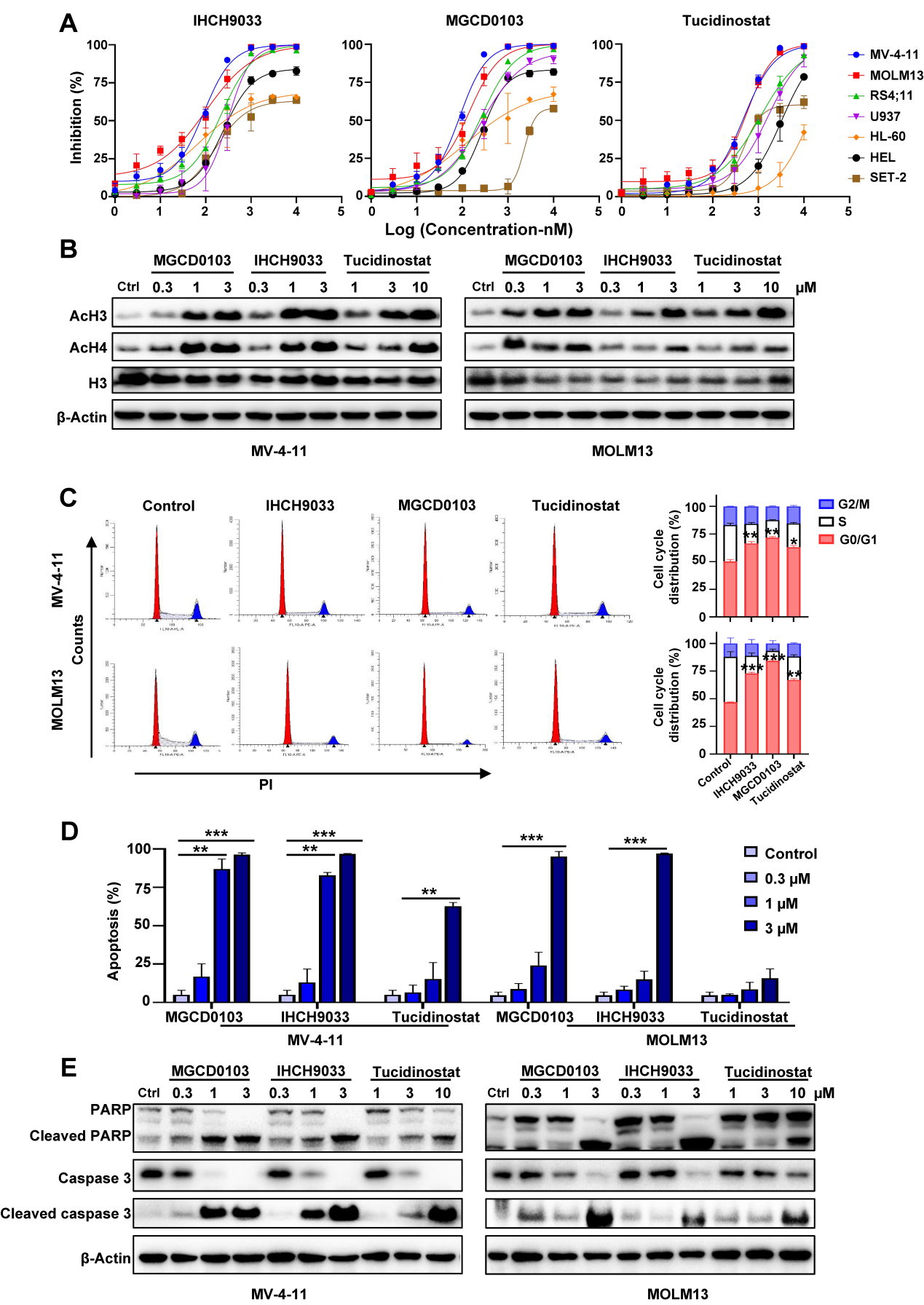
**

**Fig. S1** **Class Ⅰ HDAC inhibitors show potent activity in FLT3-ITD AML cells.** (A) The cytotoxicity of class I HDAC inhibitors (IHCH9033, MGCD0103 and Tucidinostat) in AML cell lines for 72 hours were tested by MTT assay. (B) Western blot analysis of expression levels for AcH3, AcH4, and H3 after MV-4-11 and MOLM13 cells treated with increasing concentrations of IHCH9033, MGCD0103, or tucidinostat for 24 hours. (C) MV-4-11 and MOLM13 cells were treated with of 1 μM IHCH9033, MGCD0103, or tucidinostat for 24 hours, and cell cycle distribution was detected by flow cytometry. (D) MV-4-11 and MOLM13 cells were treated with increasing concentrations of IHCH9033, MGCD0103, or tucidinostat for 48 hours, and cell apoptosis was detected by flow cytometry. (E) Protein levels of PARP, cleaved PARP, caspase 3 and cleaved caspase 3 were measured by western blot. Data are mean ± SD. *P < 0.05; **P < 0.01; ***P < 0.001.

**
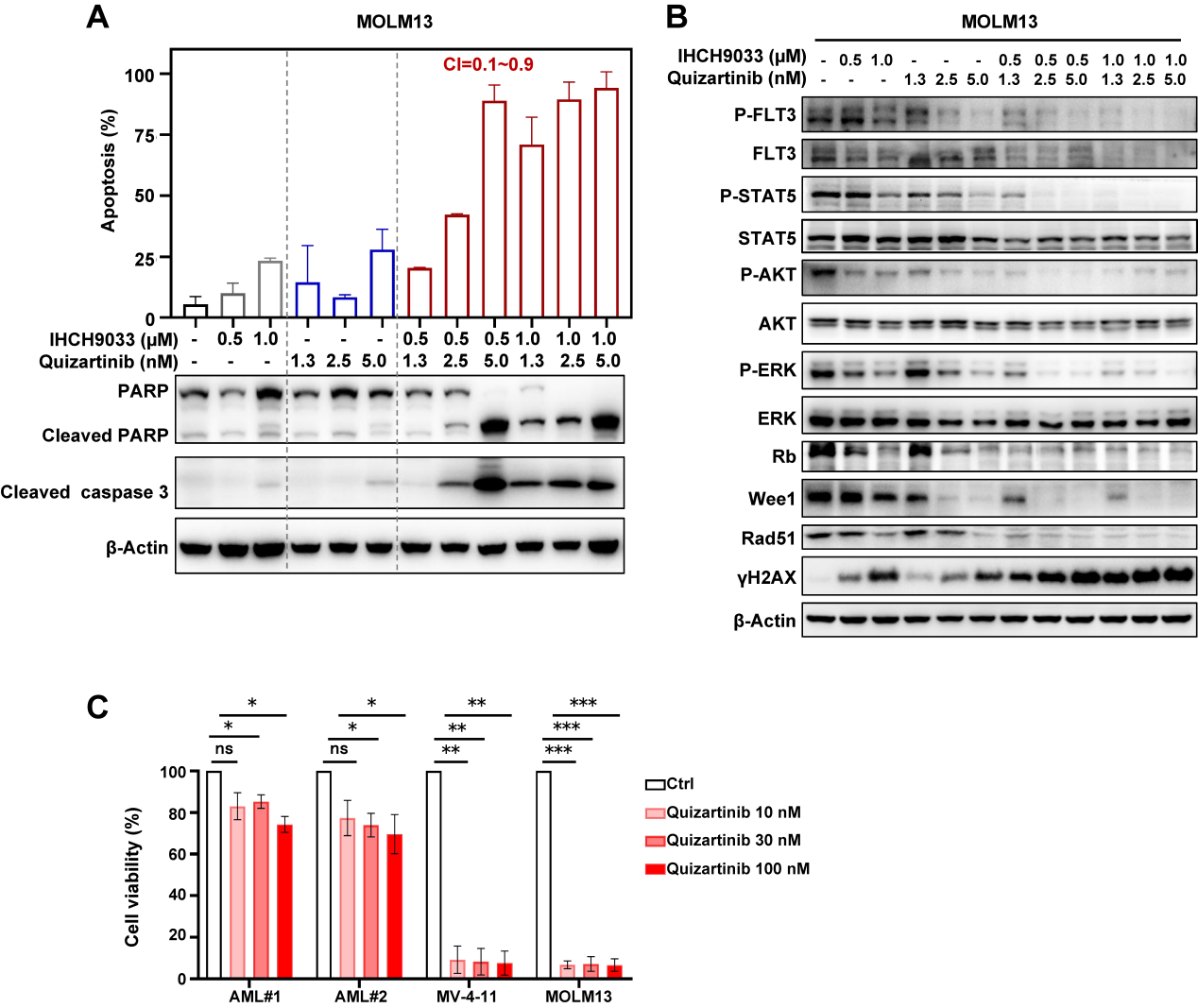
**

**Fig. S2 Combination of IHCH9033 and quizartinib synergistically induces antitumor activity in FLT3-ITD AML cells.** (A) MOLM13 cells were treated with indicated concentrations of IHCH9033, quizartinib or their combination for 48 hours, and cell apoptosis was detected by flow cytometry. Protein levels of PARP, cleaved PARP, caspase 3 and cleaved caspase 3 were measured by western blot. (B) Western blot analysis of indicated protein in MOLM13 cells treated with IHCH9033, quizartinib or their combination for 24 hours. (C) Cell viability was assessed in primary AML blasts treated with the indicated concentrations of quizartinib for 5 days, as well as in MV-4-11 and MOLM13 treated with the indicated concentrations of quizartinib for 3 days. Data are mean ± SD. *P < 0.05; **P < 0.01; ***P < 0.001.

**
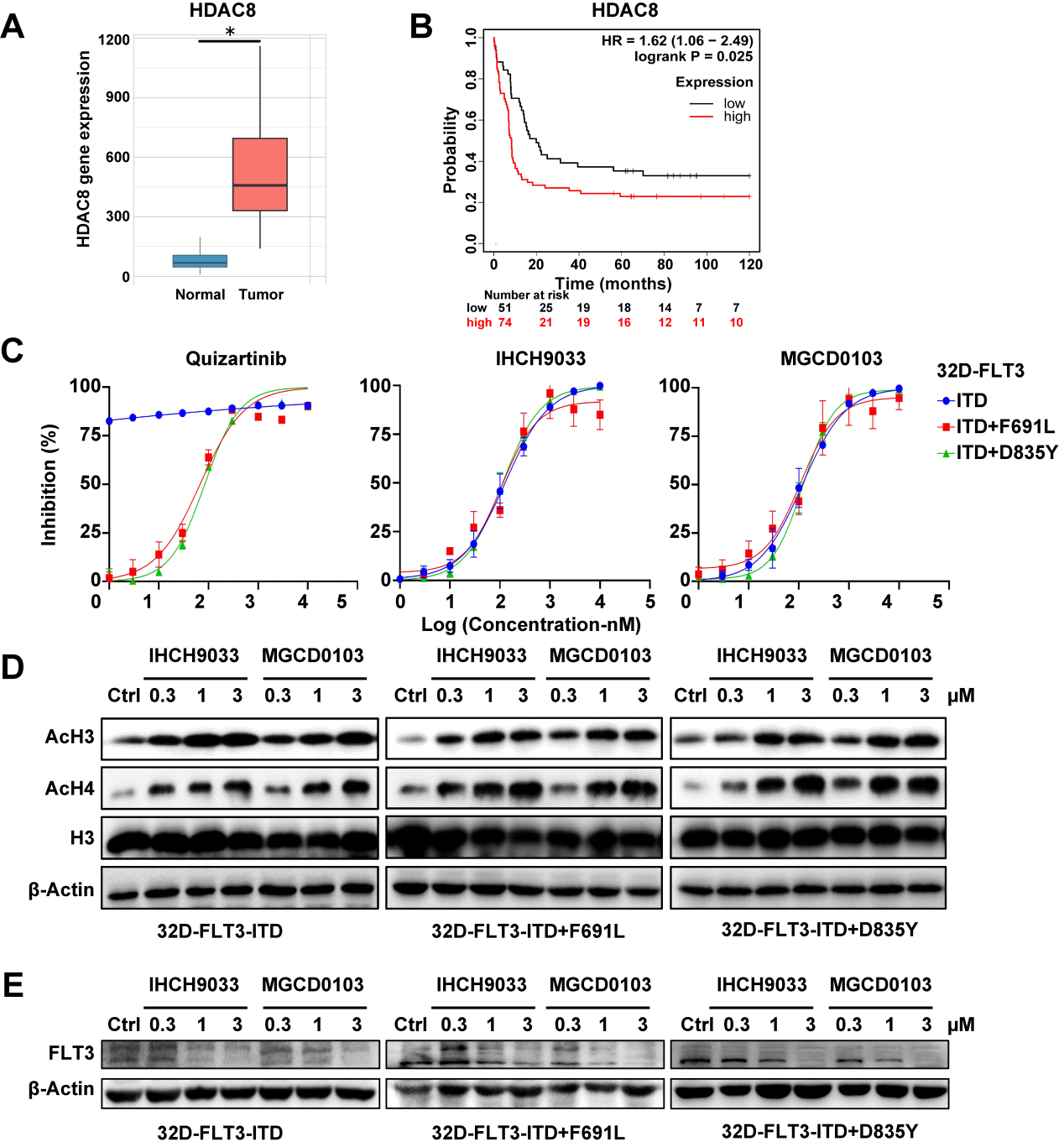
**

**Fig. S3 The class I HDACis exhibit its potency against AML, even in cases of FLT3 inhibitor resistance.** (A) Gene expression analysis of *HDAC8* in AML and normal tissues from TNMplot database (http://www.tnmplot.com). (B) Survival analysis using Kaplan–Meier estimators and the log-rank test. Differences in survival times were analyzed by comparing FLT3 AML patients with high vs. low levels of HDAC8. (C) Proliferation of 32D cells harboring FLT3-ITD/TKD (FLT3-ITD, FLT3-ITD+D835Y or FLT3-ITD+F691L) was determined after treatment with quizartinib, IHCH9033, or MGCD0103 for 72 hours using MTT assay. Data are presented as mean ± SD (n=3). (D) Acetylation levels of histone H3 and histone H4, as well as (E) the expression level of FLT3 were detected by western blot analysis after treatment with IHCH9033 or MGCD0103 for 24 hours in 32D cells harboring FLT3-ITD/TKD.


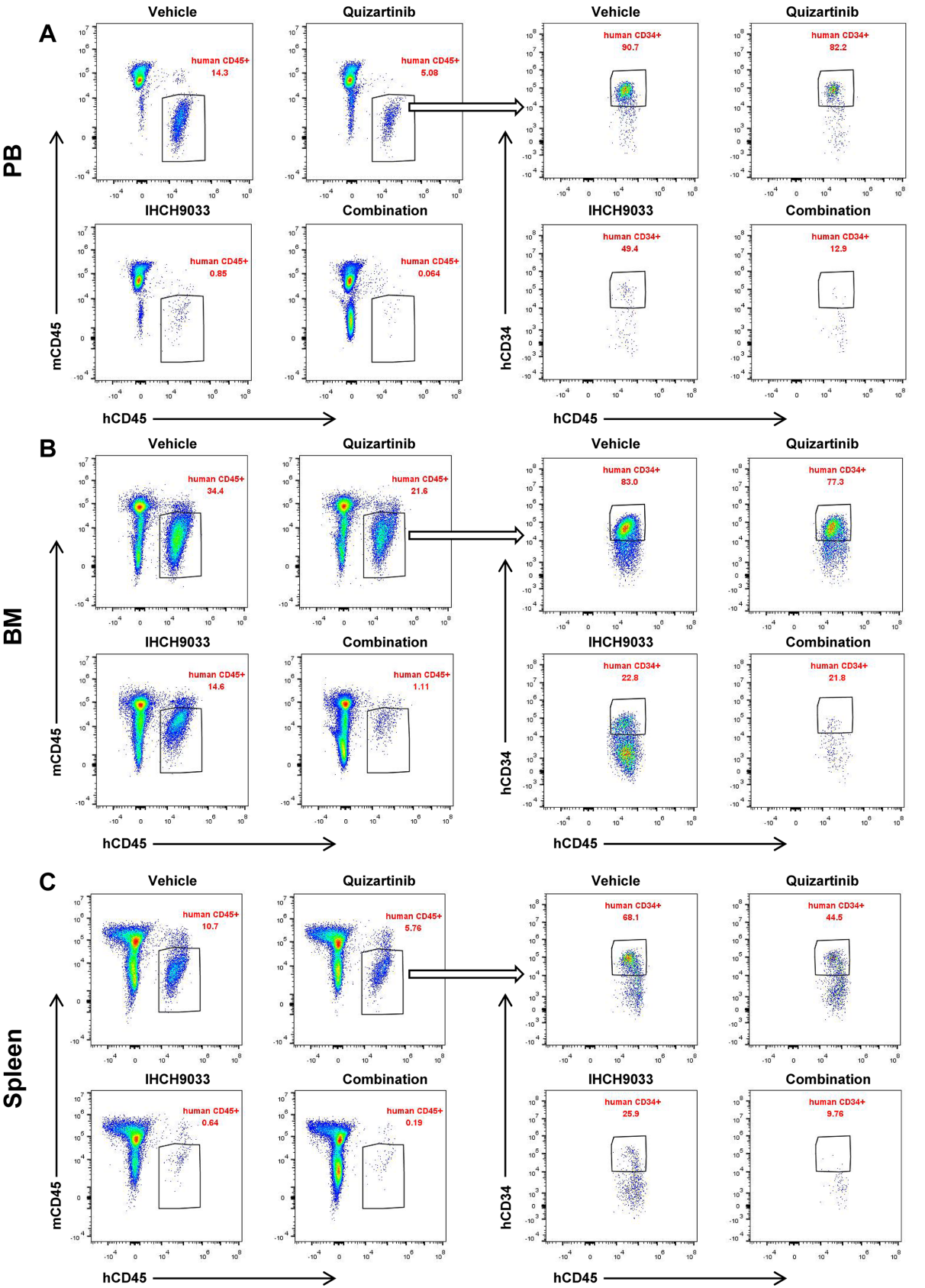


**Fig. S4 The combination of IHCH9033 and quizartinib reduces tumor burden in a FLT3-ITD AML PDX model.** NOG mice were engrafted with primary FLT3-ITD leukemia samples, after which groups of mice (n = 3-4) were treated with vehicle, IHCH9033 (60 mg/kg, every two days), quizartinib (10 mg/kg, qd), or their combination for 4 weeks. (A-C) Engraftment in PB (A), BM (B), and spleen (C) on day 28 was assessed by determining the percentage of hCD45+ cells and hCD45+CD34+ cells, and representative data are shown.
